# Different Cortical Dynamics in Face and Body Perception: An MEG study

**DOI:** 10.1371/journal.pone.0071408

**Published:** 2013-09-06

**Authors:** Hanneke K. M. Meeren, Beatrice de Gelder, Seppo P. Ahlfors, Matti S. Hämäläinen, Nouchine Hadjikhani

**Affiliations:** 1 Cognitive Neuroscience, Tilburg University, Tilburg, The Netherlands; 2 Athinoula A. Martinos Center for Biomedical Imaging, Massachusetts General Hospital/Harvard Medical School, Charlestown, Massachusetts, United States of America; University of Leuven, Belgium

## Abstract

Evidence from functional neuroimaging indicates that visual perception of human faces and bodies is carried out by distributed networks of face and body-sensitive areas in the occipito-temporal cortex. However, the dynamics of activity in these areas, needed to understand their respective functional roles, are still largely unknown. We monitored brain activity with millisecond time resolution by recording magnetoencephalographic (MEG) responses while participants viewed photographs of faces, bodies, and control stimuli. The cortical activity underlying the evoked responses was estimated with anatomically-constrained noise-normalised minimum-norm estimate and statistically analysed with spatiotemporal cluster analysis.

Our findings point to distinct spatiotemporal organization of the neural systems for face and body perception. Face-selective cortical currents were found at early latencies (120–200 ms) in a widespread occipito-temporal network including the ventral temporal cortex (VTC). In contrast, early body-related responses were confined to the lateral occipito-temporal cortex (LOTC). These were followed by strong sustained body-selective responses in the orbitofrontal cortex from 200–700 ms, and in the lateral temporal cortex and VTC after 500 ms latency. Our data suggest that the VTC region has a key role in the early processing of faces, but not of bodies. Instead, the LOTC, which includes the extra-striate body area (EBA), appears the dominant area for early body perception, whereas the VTC contributes to late and post-perceptual processing.

## Introduction

The visual perception of faces and bodies is crucial for successful social interaction between human beings as these two biological stimuli convey vital information on the gender, age, identity, mood, emotions, actions, and intentions of the other person. Despite the large visual differences between faces and bodies, their perception appears to rely on similar processing routines, as evidenced by the existence of a stimulus inversion effect [1] and automatic attention capturing [2], [3].

Neuroimaging studies have revealed that face processing relies on a distributed cortical network with specialized cortical areas in the occipito-temporal lobe. An area in mid-fusiform cortex, called the “fusiform face area” (FFA), is robustly and selectively responsive to faces [4], [5], [6], [7], [8]. Other important nodes of the network related to face processing include the inferior occipital gyrus (“occipital face area”, OFA) [7], [9] and the superior temporal sulcus [6], [10].

The investigation of the neural mechanisms underlying human body perception only started a decade ago. Seeing whole human bodies and body parts results in distinct hemodynamic activation of a cortical area near the middle occipital gyrus/middle temporal gyrus called the “extrastriate body area” (EBA) [11], [12], [13], [14], [15]. In addition, an area in the mid-fusiform cortex termed the “fusiform body area” (FBA) [14], [15], [16], [17], [18] appears to become active when whole bodies (both realistic and schematic) are perceived. This latter area shows substantial spatial overlap with the FFA (see de Gelder et al. [19] for an overview). Thus it appears that the perception of faces and bodies is partly based upon the same dedicated neural structures.

Despite the growing number of functional magnetic resonance imaging (fMRI) studies on body perception, the exact roles of the EBA and the FBA in body processing remain unclear. In order to distinguish between different processing stages, neurofunctional models of body processing need to incorporate information on the exact time course of activation in these face and body-selective areas. Transcranial magnetic stimulation (TMS) studies have already demonstrated the early involvement of the OFA in face processing and of the EBA in body processing [20], [21], [22]. Unfortunately however, TMS cannot target ventral temporal areas to investigate the time courses of the FFA and FBA. At present, electrophysiological data on body processing are very sparse compared to the wealth of temporal information available on face processing, and the time courses of the EBA and of the FBA are far from clear. For example, because of the spatial overlap between the FFA and FBA, it is often implicitly suggested that the fusiform gyrus has a role in body processing similar to that in face processing, assuming that encoding in EBA precedes that in FBA. However, to date no electrophysiological data exists to support this view.

Recordings from intracranial electrodes in epileptic patients during face processing have shown a face-selective component peaking around 200 ms after stimulus onset in the ventral temporal region including the fusiform gyrus (FG), and a lateral region of the temporal lobe including the middle and inferior temporal gyrus [23], [24], [25]. In extracranially measured electro- (EEG) and magnetoencephalogram (MEG), viewing faces elicits a prominent face-selective deflection at occipito-temporal sensors peaking around 170 ms. This so-called the N/M170 component has been functionally associated with the structural encoding of faces [26], [27], [28]. Different source localization studies have localized the current sources generating the face-selective N/M170 to different regions, i.e., to the fusiform gyrus [29], [30], [31], [32], [33], [34], [35], [36], [37], [38], [39], [40], [41], extrastriate occipital areas [40], [42], [43], lateral occipito-temporal cortex [44], lateral temporal lobe/superior temporal sulcus [29], [34], [45], lingual gyrus [46], or a combination of these areas. Hence, the results of source mapping studies are in agreement with the areas found in intracranial studies.

Similarities in electrophysiological correlates of face and body processing have been found at the EEG sensor level in event-related potentials (ERPs). The N170 component has been shown to be also elicited by whole body images [43], [47], [48], [49], [50]. In addition, this body N170 component displays the same electrophysiological inversion effect for inverted (upside down) bodies [49], [51], [52], [53], [54] as typically found for inverted faces [34], [55], [56].

It is not clear however, which cortical areas generate this body-selective extracranial N170 component. To date only two studies have attempted to localize the current sources underlying the body N/M170. Thierry and colleagues [43] found a large overlap between the distributed source estimates underlying the face and body N170 in an extensive area of the posterior extrastriate visual cortex. However, there were no indications for the involvement of the fusiform gyrus, neither in body nor in face processing. In an MEG study, Ishizu et al. [57] found equivalent current dipoles (ECDs) for the body and face M170 to be located close to each other in the lateral occipito-temporal cortex, with the body ECDs concentrated a little more posterior and dorsal (in the posterior middle temporal gyrus) than the face ECDs (in the posterior inferior temporal region). However, no sources were localized in the fusiform gyrus in this study either. The absence of fusiform sources in the previous studies may be due to limitations in the sensitivity of the source mapping approaches. As a consequence, the time courses of the EBA and FBA activity still remain elusive.

The purpose of present study is to examine the time courses of body- and face-processing related activity in the ventral temporal lobe and the lateral occipito-temporal cortex. We addressed the following questions: What are the cortical regions generating the ‘M170’ in face and body processing? What are the relative contributions of the lateral occipito-temporal cortex (LOTC) and ventral temporal cortex (VTC) to this component? What are the time courses of the LOTC and the VTC and other face- and body-selective cortical areas in face and body processing? We optimized our data acquisition and analyses methods for the detection of sources in the human ventral temporal lobe. We used the excellent temporal and relatively good spatial resolution of whole-head magnetoencephalography (MEG). We included the data of both gradiometer and magnetometer sensors to ensure that we obtain as reliable information from deeper as well as superficial sources in our source localization procedure as possible. In addition, each participant's individual MRI was used to anatomically constrain the locations and orientations of the distributed current sources in the minimum-norm estimates [58], [59]. MEG signals were recorded while participants viewed photographs of faces, bodies and houses. To reveal face- and body-*sensitive* regions we contrasted faces and bodies with their own Fourier-scrambled versions, in which the high level visual information is destroyed but the low-level characteristics are preserved. To reveal face- and body-*selective* regions we contrasted faces and bodies with the other stimulus categories.

## Materials and Methods

### Participants

Ten healthy right-handed individuals (mean age 27 years, range 24–36 years; four females) with normal or corrected to normal vision volunteered to take part in the experiment. All procedures were approved by the Massachusetts General Hospital Institutional Review Board, and informed written consent was obtained from each participant. The study was performed in accordance with the Declaration of Helsinki.

### Stimuli

**Figure 1:**
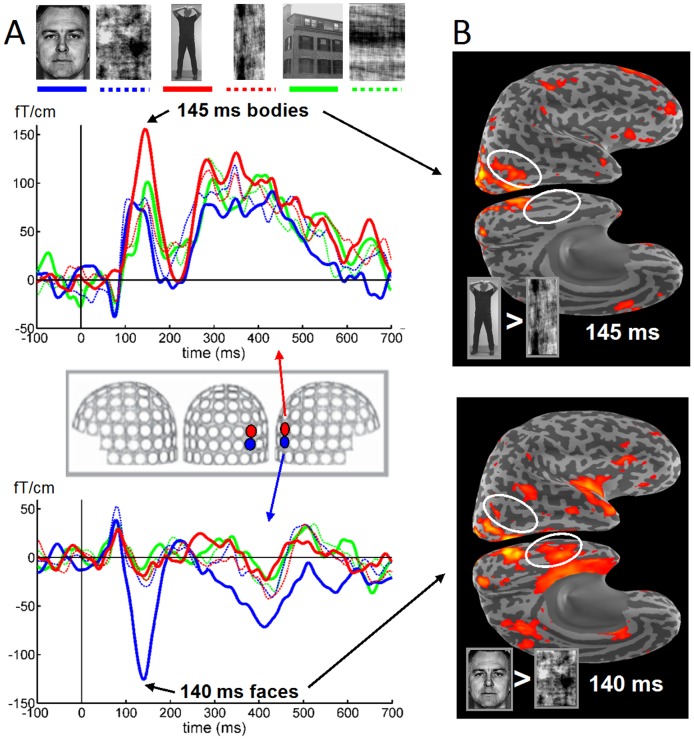
Visually evoked magnetic fields to Faces and Bodies and the corresponding estimates of cortical activity. Visually evoked magnetic fields to photographs of Upright (solid) and Scrambled (dashed) Faces (blue), Bodies (red) and Houses (green) in a representative individual. **A**. Time courses of the magnetoencephalographic (MEG) responses recorded from two adjacent lateral posterior planar gradiometers (depicted in the inset) which show different category-sensitivity around 150 ms post-stimulus for Bodies (top waveforms) and Faces (bottom), with all other categories evoking smaller responses. Examples of the stimuli used are shown at the top. **B**. Anatomically-constrained distributed MEG source estimates for Bodies and Faces at their peak latencies, visualized on the inflated cortical surface of the right hemisphere (for explanation see Figure 3F) of the same individual. Visualized are those locations where the estimated activity of Bodies (top) and Faces (bottom) is stronger than that of their Fourier phase-scrambled counterparts, with the difference in dSPM values >2. The circles point out two important differences between Bodies and Faces, with Body-related activity in the right lateral occipitotemporal lobe and Face-related activity at the ventral aspect of the temporal lobe.

Face stimuli were gray-scale photographs from the Ekman and Friesen database [60]. Eight identities (4 male, 4 females) were used, each with a neutral facial expression. Body stimuli were taken from our own validated dataset, previously used in behavioral [61], EEG [49] and fMRI studies [16], [62]. These consisted of gray-scale images of whole bodies (4 males, 4 females) adopting a neutral instrumental posture in which the faces were made invisible (for details, see Hadjikhani and de Gelder [16]). Houses constitute a familiar object category often used as a contrast to study face-selective activity [29]. The house stimuli were gray-scale photographs taken from eight different real-life brick-stone houses, with prominent orientation cues such as a roof, a door, doorsteps, part of a sidewalk, or garden. The stimuli were processed with photo-editing software in order to equalize contrast, brightness, and average luminance. We employed a Fourier-phase scrambling procedure to convert the original images into incoherent non-object images in which low level features such as overall luminance and spatial frequencies are preserved. This is important as early MEG responses are sensitive to the physical characteristics of the stimulus [63]. After randomizing the phases using a two-dimensional Fast Fourier Transform, scrambled images were constructed using the original amplitude spectrum. All images (photographs and scrambles) were pasted into a gray square (with an equal average gray value as the photographs), such that the final size of all stimuli was the same. Examples of the stimuli can be found in Figure 1.

### Experimental Design

The experiment comprised a stimulus orientation judgment task in which participants were shown 6 different stimulus categories, *i.e.*, intact faces, bodies, and houses, as well as their scrambled versions. The intact stimuli were shown in an upright and upside down (inverted condition) orientation, thus summing up to 9 stimulus conditions. Part of the data of the present experiment has previously been used to assess early (<100 ms latency) category-selective cortical activation by contrasting upright and inverted images [64]. In the present paper we focus on the responses after 100 ms after stimulus onset. The experiment was divided into four blocks each consisting of 288 trials. Within one block, all stimuli (8 exemplars * 9 stimulus conditions) were presented 4 times in random order, summing up to a total of 128 trials for each stimulus condition over the four blocks (8 exemplars * 4 random repetitions * 4 blocks). Half of the subjects started responding with their left hand, while the other half started with their right hand. At each new block the participants changed the button box to their other hand. To familiarize the subjects with the procedure and task demands the experiment was preceded by a short training session, which contained all stimulus categories.

The experiment was conducted in a magnetically shielded room (Imedco AG, Hägendorf, Switzerland). Subjects were comfortably seated with the head leaning against the back of the helmet of the MEG dewar. The visual stimuli were presented with a LP350 Digital Light Processor projector (InFocus, Wilsonville, OR) onto a back-projection screen placed 1.5 m in front of the subject. The size of the framed stimuli on the screen was 17×17 cm, subtending a visual angle of 6.5°. The stimuli were presented for 250 ms with an interstimulus interval that ranged between 2500–3000 ms. The stimuli were preceded and followed by a black fixation cross on a gray background, presented for 1000–1500 ms pre-stimulus and 500 ms post-stimulus. The post-stimulus fixation was followed by a screen with the word “PRESS” (1000-ms duration) prompting subjects to respond by an appropriate button press. The participants' task was to keep their eyes fixed on the cross and to indicate whether the picture was presented Upright, Inverted, or Scrambled. In addition, they were instructed to minimize eye blinks and all other movements. Only upright and scrambled trials were analysed for this study.

### MEG Data Acquisition

The MEG data were acquired with a 306-channel Neuromag VectorView system (Elekta-Neuromag Oy, Helsinki, Finland), which combines the focal sensitivity of 204 first-order planar gradiometers with the widespread sensitivity of 102 magnetometers. Eye movements and blinks were monitored with vertical and horizontal electro-oculogram. The location of the head with respect to the sensors was determined using four head-position indicator coils attached to the scalp. A head-based MEG coordinate frame was established by locating fiduciary landmarks (nasion and preauricular points) with a Fastrak 3D digitizer (Polhemus, Colchester, VT). The data were digitized at 600 samples/second with an anti-aliasing low-pass filter set at 200 Hz.

MEG signals were averaged across trials for each condition, time-locked to the onset of the stimulus. A 34-ms delay between the time the computer sent an image and the time it was projected onto the screen was measured with a photodiode and subsequently taken into account when reporting the timing of measured activity. A 200-ms pre-stimulus period served as baseline. Trials to which subjects made an incorrect response and those that contained eye blinks exceeding 150 µV in peak-to-peak amplitude or other artifacts were discarded from the average. The evoked responses were low-pass filtered at 40 Hz.

### Structural magnetic resonance imaging (MRI)

MEG data were co-registered with structural high-resolution magnetic resonance images (MRI). A set of 3-D T1-weighted MR images using a 1.5 T system were acquired. The MRI and MEG coordinate systems were aligned by identifying the fiducial point locations in the MRIs. In addition several points were digitized from the head surface to allow confirmation and fine tuning of the initial alignment based on the fiducial landmarks.

The geometry of the cortical mantle was extracted from the MRI data using the Freesurfer software [65], [66]. An inflated representation of the cortical surface was used for visualization to allow viewing the gyral pattern and the cortex embedded in fissures. The cortical gyri and sulci were automatically parcellated using the Freesurfer software [67], [68].

### Global MEG measures

MEG data was first quantified at the sensor level. The mean global field power (MGFP) was calculated separately for the magnetometers and the gradiometers by squaring the signal values and averaging them across sensors. Statistical group analysis was performed by one-sided *t*-tests for paired samples (*α* = 0.05) on the MGFP at successive time points.

### MEG Source Estimation

The source current distribution was estimated at each cortical location using a depth-weigthed the minimum-norm estimate (MNE) [58], [69]. The cortical surface was sampled with ca. 5000–7000 dipoles at the interface between gray and white matter provided by Freesurfer with an average 7-mm spacing between adjacent source locations. The forward solution for each of the three dipole components at each of these locations was computed for all 306 sensors using an anatomically realistic single-compartment Boundary-Element Model [70]. The inner skull boundary for this model was derived from each subject's MRI. The strength of the fixed-location sources was estimated for each time-instant of the evoked response applying the linear inverse solution using a cortical loose orientation constraint [71].

The resulting current amplitudes were noise-normalized by dividing the magnitude of the estimated currents at each location by their respective standard deviations [59]. The latter was estimated with help of the spatial noise-covariance matrix, which was computed from the 200-ms pre-stimulus activity in the non-averaged data set with the same filter settings as for the evoked responses. This noise-normalization procedure reduces the location bias towards superficial currents, inherent in the minimum-norm solution, and equalizes the point-spread function across cortical locations [59]. The noise-normalized solution provides a dynamical Statistical Parametric Map (dSPM), which essentially indicates the signal-to-noise ratio of the current estimate at each cortical location as a function of time. Thus, dSPM movies of brain activity are useful for visualization of the data as they identify locations where the MNE amplitudes are above the noise level.

*Group movies* were created by morphing the source estimates for each individual subject to the cortex of one representative subject, according to the method of Fischl et al. [72]. Subsequently, the values were averaged across individuals at each source location. The dSPM values were used to identify spatiotemporal cortical patterns that show consistent responses across individuals.

#### Statistical Analysis

Prior to statistical testing, the cortical surface of each individual subject was spatially down-sampled to 642 dipoles per hemisphere by morphing its source estimates to the cortex of one representative subject, according to the method of Fischl et al. [72]. In order to make statistical inferences on the source level we tested the depth-weighted MNE values for significant differences between the intact and scrambled conditions and among the intact conditions (e.g. intact faces>scrambled faces; intact bodies><intact faces) across subjects (random effects). Nonparametric randomization tests based on spatiotemporal clustering [73] were performed using the “FieldTrip” open-source toolbox [74] and custom software. By clustering neighboring cortical locations and subsequent time points that show the same effect, this test deals with the multiple comparisons problem in both space and time while taking into account the dependency of the data [73]. First, for each cortical location-time sample a paired-samples t-value was computed testing the intact-scrambled contrast >0 (*n* = 10, *df* = 9, *α* = 0.05, one-sided) or testing the contrast between two different stimulus categories (*n* = 10, *df* = 9, *α* = 0.05, two-sided). Second, all samples were selected for which this t-value exceeded an a priori threshold (uncorrected p<0.05). Third, the selected samples were clustered on the basis of spatial and temporal adjacency. Cortical dipoles were considered to be neighbors if their distance was less than 12 mm. A sample was only included into the cluster when there were at least two neighboring samples in space or time. Fourth, the sum of the t-values within each cluster was used as cluster-level statistic. The cluster with the maximum sum was used as test statistic. Fifth, a reference distribution of the test statistic was obtained by randomizing the data across the two conditions and recalculating the maximum cluster t-value a thousand times. Sixth, the null hypothesis of no difference between conditions was tested by evaluating the test statistic of the observed data against this reference distribution.

### Time courses from regions of interest (ROIs)

We investigated the time courses of the source data of several regions of interest (ROIs). As the MNE is a solution to an underdetermined inverse problem, in our case with 306 measurements (sensors) at each time point, and about 7000 unknown dipole elements, the interpretation of the localization of activation should be taken with caution. To test our hypotheses concerning the contribution of different lateral and ventral occipito-temporal sources to the generation of the ERF components on a coarse spatial scale, we selected four large cortical regions on basis of our *a priori* hypothesis: the full lateral aspect of the occipital lobe (LOC, which includes the functional OFA), the full lateral aspect of the temporal lobe (LTC), the entire ventral aspect of the temporal lobe (VTC, which includes the functional FFA and FBA) and the lateral occipito-temporal cortex (LOTC, which includes the functional EBA). The latter area covered the lateral surfaces of the anterior part of the occipital cortex and the posterior part of the lateral temporal cortex. These anatomical regions were defined in each individual's reconstructed cortex by merging the labels of relevant gyri and sulci which resulted from the automatic parcellation procedure [67], [68]. The anatomical validity of the labels was checked with the aid of the atlas of Ono et al. [75].

Statistical differences in time courses between conditions were computed using the depth-weighted MNE values rather than the dSPM values. For each time sample, one-sided (category sensitivity) and two-sided (category selectivity) *t*-tests for paired samples (*n* = 10, *df* = 9) were performed on the mean current strengths across all dipoles within the region.

## Results

### Evoked Responses at the Sensor Level

The event-related magnetic fields (ERFs) of all subjects showed at least two prominent deflections, peaking at around 100 ms and around 140 ms after the presentation of the visual stimuli in the posterior sensors, which we will label as the M100 and M140 component (often also labeled M1 and M2 by others). The M100 response started to rise at 45–60 ms in gradiometers over the midline occipital region, and peaked at 80–110 ms. This midline occipital component was smaller for the intact stimuli than for their scrambled versions, but this will not be the focus of the present paper. The M100 component was followed by a second prominent deflection over lateral occipito-temporal sensors, peaking between 100–180 ms and hence labeled the M140 component. This component was larger for the intact stimuli than for their scrambled counterparts. The amplitude and spatial distribution of the M140 component showed category-selectivity, with neighboring sensors showing different responses to Faces and Bodies (Figure 1). In this early time window both Faces and Bodies, but not Houses, evoked more prominent responses in the right than in the left hemisphere sensors. The responses to Faces were in general of higher amplitude and were more widespread across the gradiometer sensors than the responses to other categories; prominent responses to Bodies were mostly restricted to one or two right posterior sensors.

### Mean Global Field Power

**Figure 2:**
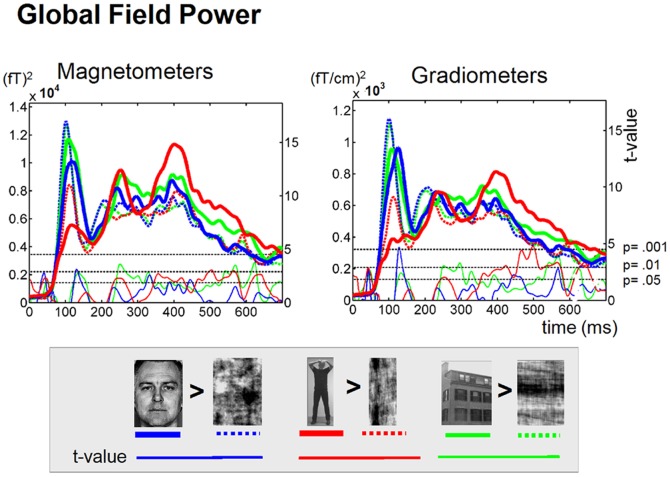
Mean global field power of face and body evoked MEG activity. The grand average (*n* = 10 subjects) of the mean Global Field Power (GFP) of the magnetometer and planar gradiometer sensors evoked by photographs of intact (solid lines) Faces (blue), Bodies (red) and Houses (green) and their Scrambled counterparts (dotted lines). Shown are the mean current strength (thick Lines) for the single conditions and the corresponding t-values (thin lines) for the contrast Intact>Scrambled (paired t-tests, one-sided, *n* = 10 subjects, *df* = 9) with color corresponding to category (Faces = blue; Bodies = red; Houses = green). The dotted black horizontal lines indicate the t-levels that correspond to the p-values of 0.05 and 0.01 and 0.001.

As a first quantitative exploration of our data we compared the overall signal magnitude at the sensor level between intact and scrambled stimuli. Figure 2 shows the mean global field power (MGFP) for the magnetometers and gradiometers, averaged across all subjects. There was a prominent difference in time course of the MGFP between categories: The MGFP of Faces peaks early, around 115 ms latency, whereas the MGFP of Bodies reaches its maximum around 400 ms after picture onset. Face- and body-sensitive processing was examined by contrasting the intact upright Face and Body stimuli with their own scrambled versions. The MGFP revealed three transient periods of face-sensitive processing with the strongest effect found between 115–140 ms (*p*<0.001, *n* = 10, *df* = 9). Several periods of body-sensitive processing could be distinguished with transient activation around 250 ms (*p*<0.05) and a strong sustained effect between 350–650 ms (*p*<0.001). In contrast, during the early time window between 80–120 ms the scrambled images evoked larger MGFP responses than the intact images.

### Evoked Responses at the Source Level

We used anatomically-constrained source modeling to estimate the source current distributions for the MEG signals throughout the whole cortex with millisecond-resolution (Dale et al., 2000). Within the first 120 ms the responses to the three intact stimulus categories roughly followed the same overall spatiotemporal activation pattern. The dynamic Statistical Parametric Maps (dSPM) for the intact stimuli were consistent with activation starting focally in the medial surface of the occipital pole ∼60 ms after the stimulus onset and spreading out rapidly to more anterior, inferior, and lateral areas within the first 120 ms. The grand average dSPM in the medial occipital cortex peaked around 100 ms (M100, see also Meeren et al., [64]).

**Figure 3:**
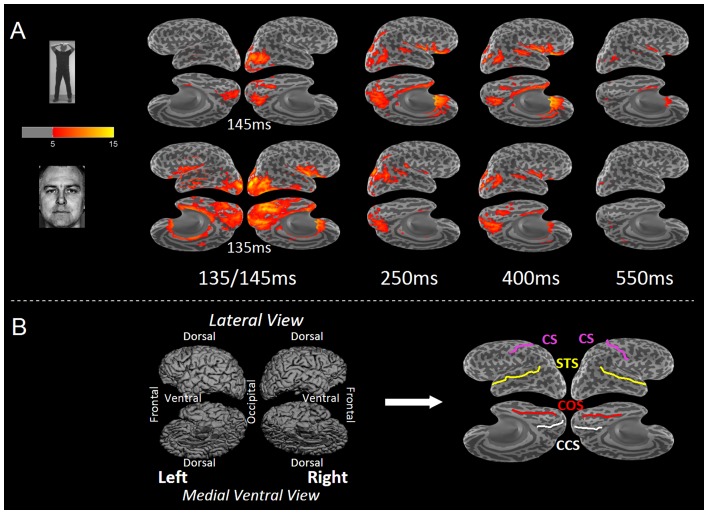
Cortical source distribution during face and body perception. Group (n = 10 subjects) results of the anatomically constrained distributed source analysis (dSPM) for Face and Body perception, visualized on the inflated cortical surface at different time instants. The first column shows the source distribution at the latency of the M140 peak response in the lateral posterior sensors (135 ms for Faces, 145 ms for Bodies). The second, third and fourth column show the source distribution at the latencies of GFP maxima for bodies at 250 ms, 400 ms and 550 ms. **A** The dSPM values for the evoked responses elicited by intact Bodies (top row) and Faces (bottom). **B**. Folded cortical maps and their corresponding inflated cortical curvature maps. Gyri and sulci are indicated in light and dark gray respectively. Both the lateral and medial surfaces are rotated towards the ventral view (11° tilted and 45° tilted respectively) to enable the depiction of the entire cerebral cortex in one quadruplet of surfaces. Major sulci are marked in the inflated maps as anatomical reference points. CS = central sulcus; STS = superior temporal gyrus; COS = collateral sulcus; CCS = calcarine sulcus.

Within the next 60 ms (120–180 ms after stimulus onset) a second wave of activation was visible spreading from posterior to anterior through the occipito-temporal lobe. This occipito-temporal response that peaked around 140 ms (M140) showed a category-specific distribution (see Figure 3). The cortical currents for bodies were strongest in the right posterior lateral occipito-temporal cortex (LOTC), while the sources for faces were much more widely distributed, i.e., throughout the medial, lateral and ventral occipital cortex, and the ventral temporal cortex.

This category-specific spatial distribution became more clear when the cortical maps elicited by the intact face and body stimuli were contrasted with the cortical maps elicited by their own Fourier phase-scrambled versions (see Figure S1). Compared with the scrambled images, faces differentially activated the entire occipital cortex (medial, lateral and ventral aspect) and the ventral and lateral aspects of a large part of the temporal lobe, including the cortex of the inferior occipital gyrus, and the middle and anterior fusiform gyrus, hence including the location of the FFA. Bodies, on the other hand, differentially evoked activity on the medial, ventral and lateral aspects of the occipital lobe and the posterior lateral occipito-temporal cortex (LOTC), a region that closely corresponds to the location of the EBA. No differential body activation was found in the ventral and anterior aspects of the temporal lobe. The direct contrast between Bodies and Faces (Figure S1) revealed that preferential activation for bodies was found in the right LOTC, and preferential activation for faces in the inferior part of the lateral and ventral occipital lobe, the ventral and lateral temporal lobe, the cingulate gyrus and the insula.

In later time windows a completely different pattern emerged. Around 250 ms latency (M250) both face and body activation was strongest in the dorsolateral occipito-temporal cortex. In the orbitofrontal cortex (OFC) a prominent sustained body-selectivity was found. Bodies gave preferential activation in the inferior, ventral and medial occipital cortex, dorsal parietal (IPS), and bilateral orbitofrontal cortex (Figure S1). In the late stages (M400 and M500) the perception of bodies elicited prominent activation in the OFC, and ventral and lateral aspects of the temporal lobe (including the STS and FG) and in the intraparietal sulcus. Faces elicited sensitive and selective activation in the LOTC.

### Spatiotemporal Cluster Analysis of the Cortical Current Estimates

**Figure 4:**
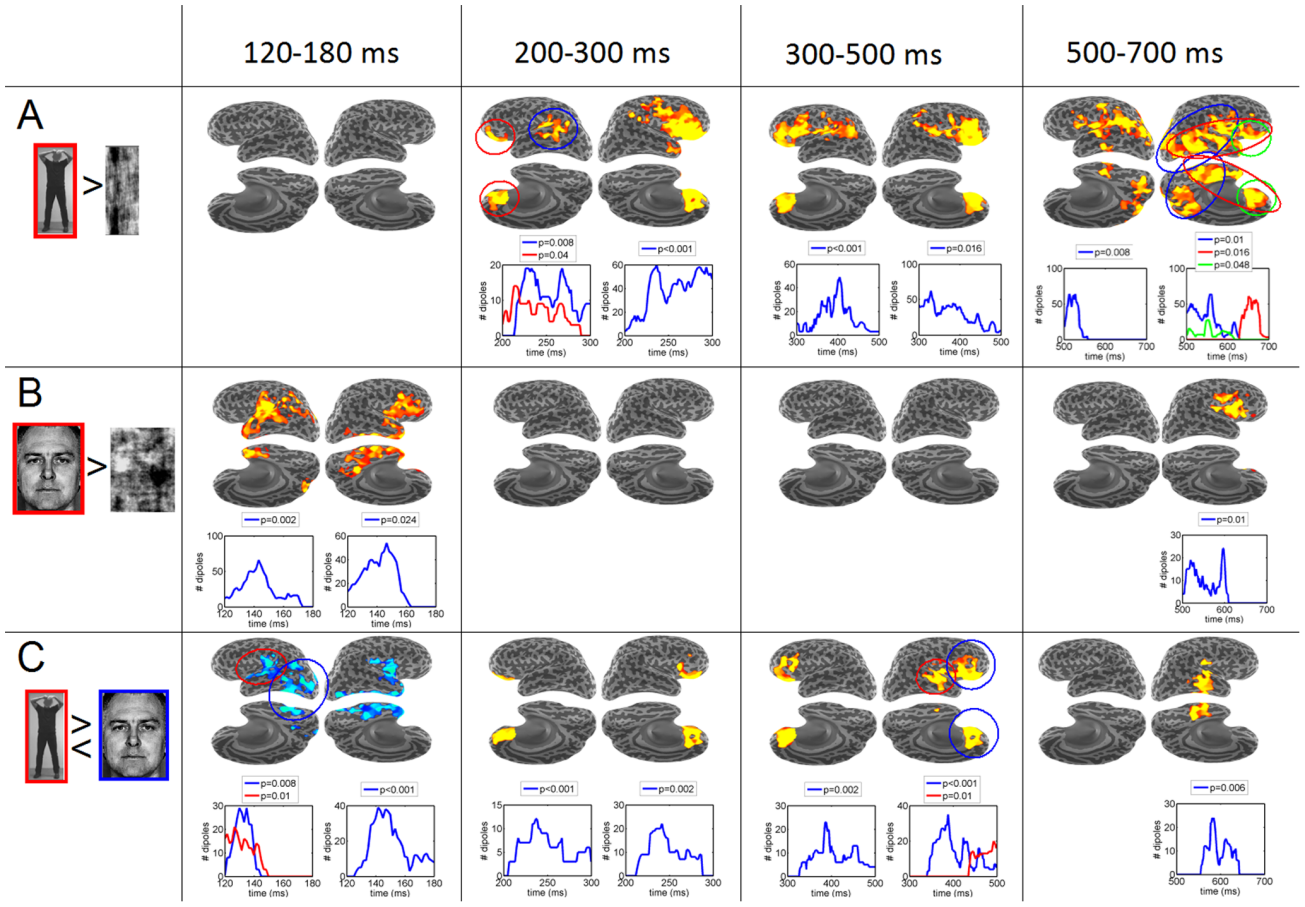
Spatiotemporal cluster analysis of cortical current estimates. Results of the spatiotemporal cluster analysis on the cortical current estimates data for Face and Body perception, visualized on the inflated cortical surface. Columns represent different time windows, rows represent different contrasts. Each cell displays both the spatial and temporal extent of each cluster. The visualization of the inflated cortical surface is equivalent to Figure 3 (see figure legend 3D), with left hemisphere on the left and right hemisphere on the right. The graphs below the cortical maps represent the temporal courses of the size of each cluster (in number of dipoles) and their p-value, in the left and right hemisphere. In the case of multiple clusters, the red, blue and green colors indicate corresponding clusters in the cortical map (circles) and time course. **A**. *Body Sensitivity* was analysed with the contrast Bodies>Scrambled Bodies (*n* = 10, one-sided, *α* = 0.05). **B**. *Face sensitivity* was analysed with the contrast Faces>Scrambled Faces (*n* = 10, one-sided, *α* = 0.05). **C**. Body and face selectivity was analysed by directly contrasting Bodies with Faces (*n* = 10, two-sided, *α* = 0.025 for each side). Clusters with preferred responses to Bodies are indicated in yellow/red; clusters with preferred responses to Faces in blue.

Figure 4 shows the results of the nonparametric spatiotemporal cluster analysis in four different time windows, i.e. 120–200, 200–300, 300–500 and 500–700 msec, with respect to body- and face-sensitivity, and body- and face-selectivity. Body and Face selective responses with respect to Houses can be found in Figure S2.

#### Body sensitivity

(Figure 4A) Body-sensitive clusters with preferred activity to Bodies compared to their Scrambled versions (i.e. Bodies>Scrambled Bodies, *n* = 10, *α* = 0.05 one-sided) were found after 200 ms latency. A large ventrolateral prefrontal – orbitofrontal cluster was active for a sustained period in the right (200–300 ms: *p*<0.001; 300–500 ms: *p* = 0.016; 500–700 ms: *p* = 0.046) and the left (200–300 and 300–500 ms: *p*<0.001) hemisphere. In addition there was significant transient activity around the left temporo-parietal junction between 200–300 ms (*p* = 0.04). The lateral occipital, lateral temporal and ventral temporal lobe showed body sensitivity during 500–700 ms.

#### Face sensitivity

(Figure 4B) Face-sensitive clusters (i.e. Faces>Scrambled Faces, *n* = 10, *α* = 0.05 one-sided) were found between 120–180 ms in the right and left hemisphere. The cluster in the right hemisphere comprised the ventral temporal cortex and right ventrolateral prefrontal cortex (*p* = 0.03). The cluster in the left hemisphere included the left temporal pole, superior aspect of the lateral temporal cortex, the insula and extended into the area of the temporo-parietal junction/angular gyrus (*p* = 0.002). In addition, a late cluster in the right hemisphere was found comprising the insula and the ventrolateral prefrontal cortex (*p* = 0.01).

#### Category selectivity

(Figure 4C and Figure S2) Differential responses between Bodies and Faces were analysed by directly contrasting the two conditions (i.e. Faces><Bodies, *n* = 10, *α* = 0.025 for each side). In the early time window of 120–200 ms Faces elicited significantly larger responses than Bodies, whereas from 200 ms onwards Bodies elicited significantly larger responses than Faces. The early face selectivity was found in the left posterior ventrolateral occipito-temporal cortex (120–140 ms, *p* = 0.024) and the left medial parietal cortex (160–200 ms, *p* = 0.02). The early face selective cluster in the right hemisphere covered the whole aspect of the VTC and part of the insula (120–180 ms, *p*<0.001).

Sustained body selectivity was found in bilateral OFC (200–500 ms, *p*<0.001). In addition, a cluster with preferred responses to Bodies was found in the right LTC (430–500 ms, *p* = 0.01). In the late time window a body selective cluster was found which covered the middle part of the ventrolateral temporal cortex (550–650 ms, *p* = 0.006).

### Time Courses of Face- and Body-Sensitive and -Selective Areas

**Figure 5:**
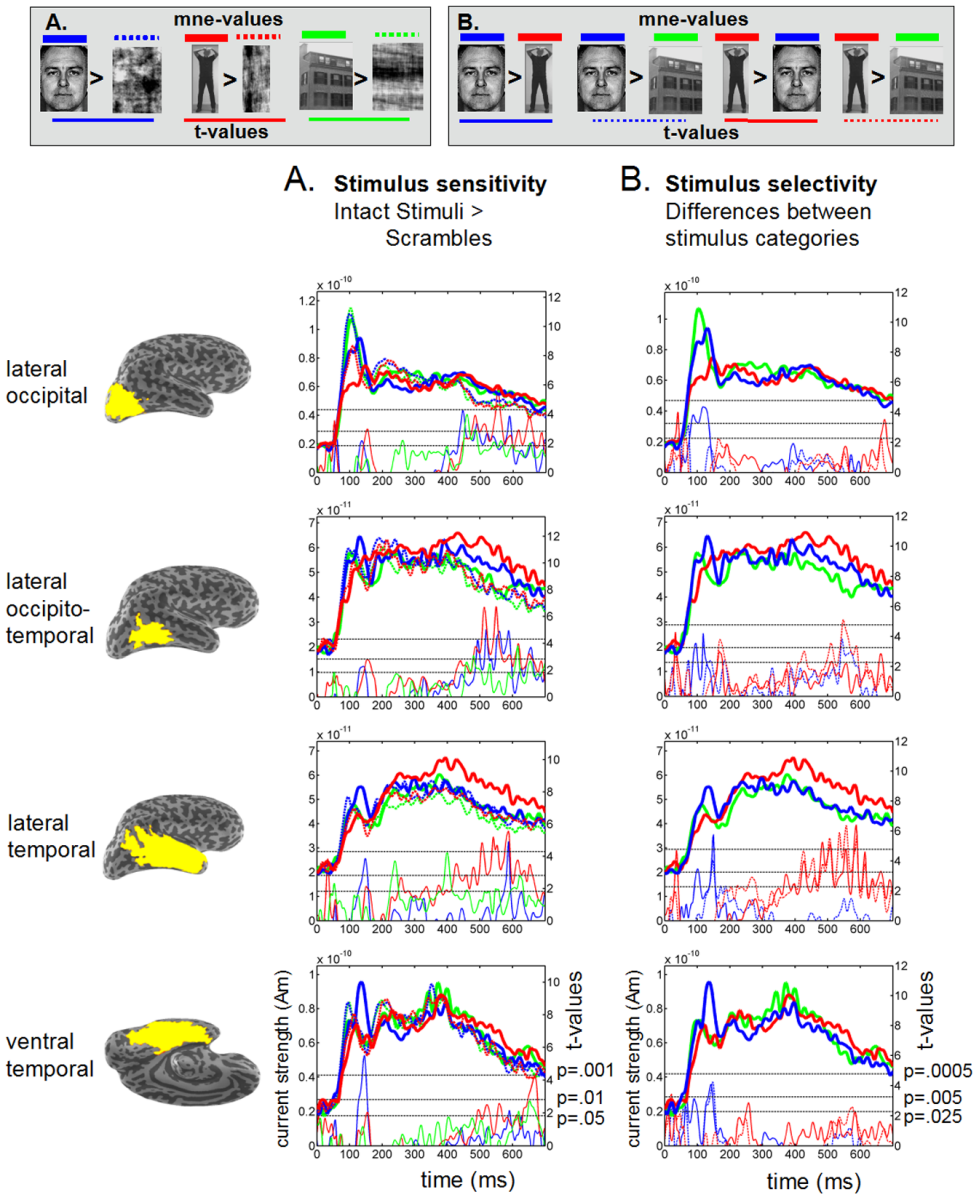
Time courses of MEG source estimates in anatomical regions of interest from the right hemisphere. Grand average (*n* = 10 subjects) time courses of the mean estimated current strength (thick lines) for intact (solid lines) Faces (blue), Bodies (red) and Houses (green) and their Fourier-scrambled versions (dashed lines), extracted from several large anatomical cortical regions. The thin line curves show the corresponding *t*-values for planned comparisons. The dotted black horizontal lines indicate the t-thresholds that correspond to *α*-values of 0.05, 0.01 and 0.001. **A**. *Category sensitivity*: The thin lines display the *t*-values for the contrasts between each intact stimulus category and its own scrambled counterpart (paired t-tests, one-sided, *n* = 10 subjects, *df* = 9) in blue (Faces>Scrambled Faces), red (Bodies>Scrambled Bodies) and green (Houses>Scrambled Houses). The dotted black horizontal lines indicate the *t*-thresholds that correspond to p-values of 0.05, 0.01 and 0.001. **B**. *Category selectivity*: The thin lines represent the t-values for the following contrasts: Faces>Bodies (blue solid line), Faces>Houses (blue dotted line), Bodies>Faces (red solid line) and Bodies>Houses (red dotted line). The contrasts were tested two-sided (*n* = 10, *df* = 9), but only one side is presented in the graph. Consequently, the p-values correspond to *α*/2. The dotted black horizontal lines indicate the t-thresholds that correspond to *p*-values of 0.025, 0.005 and 0.0005. Note that the vertical scales on the left axis for mne-values, and on the right axis for the *t*-values vary between graphs.

To investigate the time courses of category sensitivity and selectivity into more depth, we extracted the time courses of the current strengths from four large anatomical regions in the occipito-temporal cortex that were *a priori* selected on basis of their well-documented involvement in visual processing. Figure 5 and Figure S3 show the results for the right and left hemisphere of the quantitative analysis (paired t-tests) to investigate category sensitivity (Figure 5A and Figure S3A, i.e., whether the response to the intact stimulus was larger than the response to its scrambled counterpart), and category selectivity (Figure 5B and Figure S3B, i.e., whether a certain stimulus category elicited larger responses than a different stimulus category). These time courses confirm and complement the picture that emerged from the spatiotemporal statistical clustering procedure.

#### M140 activity

Faces elicited a high-amplitude M140 response in the VTC of the right hemisphere, which showed both strong face-sensitivity (Faces>Scrambled Faces; *p*<0.001) and face-selectivity (Faces>Bodies/Houses; *p*<0.005). In addition, the LTC showed face-sensitivity (*p*<0.01) and strong face–selectivity (Faces>Bodies/Houses, *p*<0.0005). All other areas except the LOTC showed a marginal face-sensitive response (*p*<0.05). Early face-selectivity was seen in all investigated regions. The analog regions in the left hemisphere (Figure S3) showed responses that were almost as strong, the main differences being stronger face-selectivity in the left LOC (p<0.0005) and weaker face-selectivity in the LTC and VTC.

The M140 elicited by bodies peaked slightly later than that of faces. In contrast to the absence of effects in the cluster analysis, the ROI analysis did show some degree of early body-sensitivity. The strongest body-sensitivity was found in the LOTC (*p*<0.01) and LOC (*p*<0.01). The only region showing early body-selectivity was the LOTC (Bodies>Houses, *p*<0.005; Bodies>Faces, *p*<0.025).

A clear house-related M140 response was absent in the right hemisphere, nor was there any sign of house-*sensitivity*. In the left hemisphere however, houses elicited relatively strong responses, showing both significant house-sensitivity and house-selectivity (*p*<0.01, Figure S3).

#### M250 activity

Faces did not evoke prominent responses in the 200–300 ms time window. No face-sensitivity or face-selectivity was found. Whereas the responses to faces attenuated after 200 ms, those to bodies increased, resulting in body-sensitive responses in the LTC (*p*<0.05). Body-*selective* responses were found in the LTC and VTC (*p*<0.025).

#### Late (>300 ms) activity

Face stimuli elicited significantly larger responses than Scrambled Faces from 400 ms onwards in the LOC (*p*<0.01) and from 500 ms onwards in the LOTC (*p*<0.001). In addition, a transient face-sensitivity could be noted around 600 ms in the LTC. Of these responses the LOTC showed the strongest face-*selectivity* with respect to houses (*p*<0.005). Late body-*sensitivity* was seen in all investigated cortical regions of both the right and the left hemisphere, but most prominently in the right LOC, LOTC and LTC (400–700 ms, *p*<0.001), the right OFC (200–700 ms, *p*<0.001), and the left VTC (500–700 ms, *p*<0.001). These responses were strongly *body-selective* compared to both faces and houses in the LTC (400–700 ms, *p*<0.0005). In addition, Body-selectivity with respect to houses was found in the LOTC (400–700 ms, *p*<0.0005).

## Discussion

We studied the neural responses related to face and body perception using MEG. In the data analysis, we employed, in addition to conventional sensor space measures, anatomically-constrained distributed source modeling combined with spatiotemporal cluster analysis. The ERFs evoked by face-stimuli differed from the ERFs evoked by body-stimuli in a number of aspects. First, whereas face ERFs showed high global field power at early latencies, i.e. large transient activity around 150 ms, body ERFs showed high global field power at later latencies, i.e. sustained activity between 200–700 ms after picture onset (see Figure 2). Second, these differences were also reflected in the estimated cortical current sources. The high-amplitude face M140 component appeared to be generated by widely distributed current sources in the bilateral occipito-temporal cortex. These sources were maximal in the occipital cortex and the posterior ventral occipito-temporal cortex, but also strong in the LOTC, VTC, LTC and Insula (see Figure 3). Of these sources, those in the right VTC, bilateral temporal pole, bilateral insula-VLPF and left parietal cortex showed *face-sensitivity*, i.e. faces eliciting larger responses than their scrambled counterparts, despite similar low-level characteristics. Moreover, the sources in the right VTC and insula, and left occipito-temporal and medial parietal cortex showed *face-selectivity*, i.e. faces eliciting larger responses than bodies and houses (see Figure 4).

In contrast to the wide distribution of sources underlying the high-amplitude face M140, the low-amplitude body M150 appeared to be generated by a much more restricted cortical area (see Figure 3). Its main contributor was located in the right LOTC, a region corresponding to the right EBA. No convincing evidence was found for *body-sensitivity* or –*selectivity* in the LOTC. Although the body-related M150 responses in LOTC showed significant differences with scrambles, faces and houses in the ROI-analysis (see Figure 5), no such differences were found with the cluster-analysis (see Figure 4).

The spatial distribution of the MEG source estimates of the M140 for faces agrees well with intracranial EEG and fMRI findings, indicating that our source estimation approach yielded physiologically meaningful results. Most of the previous EEG and MEG source analysis studies have revealed only one or two active source areas in the N/M170 time-window, either in lateral occipital regions [42], in the FG [30], [31], [32], [33], [34], [35], [36], [37], [38], lingual gyri [46], LOTC [44], or lateral temporal lobe/STS [34], [45]. In the present study, we were able to identify a whole network of cortical areas, which included many nodes of the distributed face-processing network identified previously (i.e. IOG, FG, ITG, STG, anterior STS [76], and anterior temporal lobe [77], hereby bridging a gap between ERP and fMRI results.

To date there exist only two source localization studies on the body N/M170 component [43], [57]. Using EEG, Thierry et al. [43] found a large overlap between the cortical source estimates (LAURA) of the face N170 and the body N190 in the right posterior extrastriate cortex, probably including the area of the EBA. In the present MEG study we found a similar overlap of sources for faces and bodies in the LOTC, but in addition we found face-selective sources in the ventral and lateral temporal cortex, lateral parietal cortex and insula. This extended face-selective patch was not found by Thierry et al [43]. In a previous EEG study, we also found striking similarities of ERP time course and topography for faces and bodies [49] suggesting highly similar processing routines for the two stimulus categories. It appears that MEG, compared to EEG, is more sensitive to pick up activity from deep tangential sources in the ventral temporal lobe [34].

One previous MEG study [57] found ECDs of the M170 to be located in the occipito-temporal cortex, with body ECDs concentrated in the posterior middle temporal gyrus, and face ECDs centered in the posterior inferior temporal region. However, more anterior ventral sources for faces, e.g. in the fusiform gyrus, were not reported. In their source mapping approach only signals of the planar gradiometer sensors were used. In our study, we purposely also included the data of the magnetometers, which are more sensitive for deep sources than the gradiometers, in our source localization procedure, to maximize the detectability of sources in the ventral temporal cortex. Indeed, we observed prominent activity in the ventral temporal lobe (for faces, see Figure 4A) and the ventral surface of the frontal lobe (for bodies, see Figures 3, 4 and 5), areas that are expected to yield a relatively low signal-to-noise ratio [78]. It is, however, feasible that sources in these areas can be detected by MEG [34], [40] when extended patches are activated, as the summating sources from the crown of a gyrus and bottom of a sulcus have a predominantly tangential orientation whereas the canceling sources from opposing sites of the sulcal wall are predominantly radial [79].

No evidence was found for the VTC contributing to the body M150 response in the present study. The fact that we found strong activation in the VTC for faces but not for bodies points to an important difference in functional neuro-anatomical processing of the two stimulus categories. Evidential support for our findings of the presence (face processing) and lack (body processing) of involvement of ventral sources in the generation of the M140 comes from studies which correlated hemodynamic activity with the ERP N1 component. Taylor et al. [80] found that the functional profile of the N1 component in body processing correlates with the functional profile of hemodynamic activation in the EBA but not the FBA. In contrast, simultaneous fMRI-ERP recordings during face perception have shown a high correlation between the N1 amplitude and hemodynamic activity in the FFA and pSTS but not in the OFA [81].

A third important difference between body and face ERFs is the extent of the underlying cortical sources at later latencies. Extensive body-selective cortical activation occurred in dorsal, frontal, and temporal regions, with the OFC showing significant body-selective responses after 200 ms after stimulus onset, and the VTC and LTC after 500 ms latency. In contrast, no face-sensitive or selective activity could be found between 200–500 ms. The only face-sensitivity that could be detected was noticeable after 500 ms latency in the region of the right insula-VLPF.

### Implications for neural models on face and body processing

In the present study subjects had to make an upright/inverted judgment, a task that requires to detect the stimulus category, and to analyze the configural properties of the stimuli. Under these equivalent task requirements, body and face stimuli elicited a very different pattern of cortical activation with respect to both location and timing. Whereas widespread face-related activity peaked around 140 ms post-stimulus, bodies evoked a transient activity in the LOTC peaking at 150 ms and a prominent sustained activity later in time peaking at 400 ms. These results indicate that extensive cortical resources were involved at different points in time. Viewing faces recruited a widespread distributed network of cortical areas involving early activation of the LOC, LOTC and VTC (including the functional areas of the OFA and the FFA), corresponding to the time window of the visual analysis and structural encoding of the stimulus [28], [82]. In contrast, during the same early visual perception stage, bodies activate a much more restricted area in the LOTC (including the functional area of the EBA), suggesting that the main area for the visual analysis of bodies is the EBA and not the FBA. The lack of early VTC activation in body perception, however, makes it unlikely that the FBA is involved in early body detection or the detection of first order spatial relations between body parts, as previously suggested on basis of fMRI findings [51], [53], [83], [84].

Extensive body-selective cortical activation occurred at later latencies in dorsal, frontal, and temporal regions, with the OFC showing significant body-selective responses after 200 ms after stimulus onset, and the VTC and LTC after 500 ms latency. This suggests that the hemodynamic activation of the FBA by bodies as found in fMRI studies [14], [15], [16], [17], [18] may rather reflect later stages of visual recognition and not the primary visual analysis per se. The spatial overlap between the FBA and the FFA as identified by fMRI [18], [76] has been used to argue that the role of the FBA in body processing is similar to that of the FFA in face processing [51], [53], [83], [84]. Our present results however, do not support such a similar role for face and body perception. We could not find any indication for selective VTC and LTC activation by body stimuli during the early stages. Rather, the late body-selectivity observed in the VTC suggests that the FG is involved in a variety of late perceptual processes. For example, it may reflect secondary activation of face-selective neurons (but see Peelen et al. [85]) contextual activation of the FFA by body stimuli [86], or visual imagery of the face.

Among the areas that showed a late sustained body-selective response, the OFC stands out. Time courses in these areas suggest that they are not mediating the core visual analysis of body stimuli, but rather are involved in later perceptual processes. Recently, a corresponding orbitofrontal area has also been found to respond hemodynamically to human bodies during an oddball detection task [87] and during the recognition of one's own body [83], [84]. In addition, the OFC is an important structure in the emotion-processing network, has previously been implicated in face-processing [77], [88], and is activated by stimuli with high saliency or biological relevance [89].

A few notes of caution should be made, however, interpreting the results. First, the present task of orientation judgment could have been performed on the basis of low spatial frequencies only. The FG may be involved in fine-grained analysis and higher-order spatial relationships in *both* face *and* body processing, two processes needed for identity recognition. In the present study, fine-grained face analysis and face identity may have been automatically encoded, but not body identity. This would not so much point to different functionalities of FFA and FBA, but more to the automaticity in which the FG is driven by faces as compared to bodies. The distinct spatiotemporal patterns may result from the difference in information that can be read from faces and bodies [19], [90]. When viewing faces we rapidly and automatically encode a person's identity, age, gender, and attractiveness, whereas a fully dressed human body will signal foremost the posture and (implied) actions, activating areas involved in action understanding [62], [91], [92]. Whatever the exact reason and underlying mechanism, we suggest that the (ventral) pathway to the FG is the obligatory route for faces, while other pathways are much more readily accessed for bodies (e.g., dorsal routes to frontal cortex for action recognition). In line with this, we previously showed category-selective cortical activation 70–100 ms after stimulus onset when upright and inverted stimuli were contrasted, with early dorsal stream activation (medial parietal cortex) for bodies and early ventral stream activation (IOG, mFG) for faces [64], possibly reflecting the early activation of category-selective magnocellular pathways to mediate rapid face and body detection.

Concerning the second note of caution, the present activation patterns were found for neutral faces and neutral clothed bodies with the subjects performing an orientation judgment task. It is possible that other body stimuli (e.g. bodies expressing an emotion, naked bodies) or task instructions (e.g., gender or identity recognition) may result in earlier VTC activation. In this respect it is of interest to note that naked bodies have been found to elicit a much larger N170 than clothed bodies [93].

## Conclusions

In contrast to previous EEG studies which have pointed out highly similar neural processing of face and body stimuli, the present MEG findings have also revealed clear differences between the two stimulus categories. With respect to early visual processing, the face M140 is generated by a distributed network of cortical areas in the occipito-temporal lobe, with strong contributions from the LOTC and VTC (including the FG/FFA). In contrast, the body M150 appears to be generated by a much smaller region concentrated in the LOTC, an area corresponding to the location of the EBA. No evidence was found for early activation of the VTC (including the FG/FBA) by bodies. Our MEG results suggest that the hemodynamic activation of the FBA as consistently found in fMRI studies may rather reflect late activation, i.e., after 500 ms after stimulus presentation. Hence, the current results suggest an important difference in the functional roles of cortical areas in the early analysis of faces and bodies. Whereas the FG has a prominent role in the early visual analysis of faces, our data suggests that the EBA fulfils a similar role in the early visual analysis of bodies. The role of the FG in body processing appears to be limited to late and post-perceptual processing, as indicated by the widespread late activity which included the OFC and VTC evoked by bodies.

## Supporting Information

Figure S1 **Differential maps of cortical source distribution.** Group (n = 10 subjects) results of the anatomically constrained distributed source analysis (dSPM) for Face and Body perception, visualized on the inflated cortical surface at different time instants. The first column shows the source distribution at the latency of the M140 peak response in the lateral posterior sensors (135 ms for Faces, 145 ms for Bodies). The second, third and fourth column show the source distribution at the latencies of GFP maxima for bodies at 250 ms, 400 ms and 550 ms. **A**. Regions of Body- and Face-sensitivity were explored by subtracting the dSPM values of the Scrambled bodies and faces from the dSPM values of intact Bodies and Faces. **B**. Differential dSPM maps were created by contrasting the Body and Face dSPM maps directly to each other to explore regions showing category-preferred responses. The visualization of the cortical surface is identical to that in Figure 1 of the main article. The face image was taken from Ekman & Friesen [94].
(TIF)

Figure S2 **Spatiotemporal cluster analysis of cortical current estimates with respect to Houses.** Results of the spatiotemporal cluster analysis on the cortical current estimates data for Face and Body perception, visualized on the inflated cortical surface. Columns represent different time windows, rows different contrasts. Each cell displays both the spatial and temporal extent of each cluster. The visualization of the inflated cortical surface is equivalent to Figure 3 (see figure legend 3D), with left hemisphere on the left and right hemisphere on the right. The graphs below the cortical maps represent the temporal courses of the size of each cluster (in number of dipoles) and their *p*-value, in the left and right hemisphere. In the case of multiple clusters, the red, blue and green colors indicate corresponding clusters in the cortical map (circles) and time course. **A**. Body selectivity was analysed by directly contrasting Bodies with Houses (*n* = 10, two-sided, *α* = 0.025 for each side). Clusters with preferred responses to Bodies are indicated in yellow/red; clusters with preferred responses to Houses in blue. **B**. Face selectivity was analysed by directly contrasting Faces with Houses (*n* = 10, two sided, *α* = 0.025 for each side). Clusters with preferred responses to Faces are indicated in yellow/red; clusters with preferred responses to Houses in blue. The face image was taken from Ekman & Friesen [94].
(TIF)

Figure S3 **Time courses of MEG source estimates in anatomical regions of interest from the left hemisphere.** Grand average (*n* = 10 subjects) time courses of the mean estimated current strength (thick lines) for intact (solid lines) Faces (blue), Bodies (red) and Houses (green) and their Fourier-scrambled versions (dashed lines), extracted from several large cortical regions. The thin line curves show the corresponding t-values for planned comparisons. The dotted black horizontal lines indicate the t-thresholds that correspond to *α*-values of 0.05, 0.01 and 0.001. **A**. *Category sensitivity*: The thin lines display the t-values for the contrasts between each intact stimulus category and its own scrambled counterpart (paired *t*-tests, one-sided, *n* = 10 subjects, *df* = 9) in blue (Faces>Scrambled Faces), red (Bodies>Scrambled Bodies) and green (Houses>Scrambled Houses). The dotted black horizontal lines indicate the *t*-thresholds that correspond to *p*-values of 0.05, 0.01 and 0.001. **B**. *Category selectivity*: The thin lines represent the t-values for the following contrasts: Faces>Bodies (blue solid line), Faces>Houses (blue dotted line), Bodies>Faces (red solid line) and Bodies>Houses (red dotted line). The contrasts were tested two-sided (*n* = 10, *df* = 9), but only one side is presented in the graph. Consequently, the *p*-values correspond to *α*/2. The dotted black horizontal lines indicate the t-thresholds that correspond to *p*-values of 0.025, 0.005 and 0.0005. Note that the vertical scales on the left axis for mne-values, and on the right axis for the *t*-values vary between graphs. The face image was taken from Ekman & Friesen [94].
(TIF)
