# Supplementary figures and images for: Different Cortical Dynamics in Face and Body Perception: An MEG study
Source: PLoS One. 2013 Sep 6;8(9):e71408. doi: 10.1371/journal.pone.0071408 (PMC3765413; doi:10.1371/journal.pone.0071408)

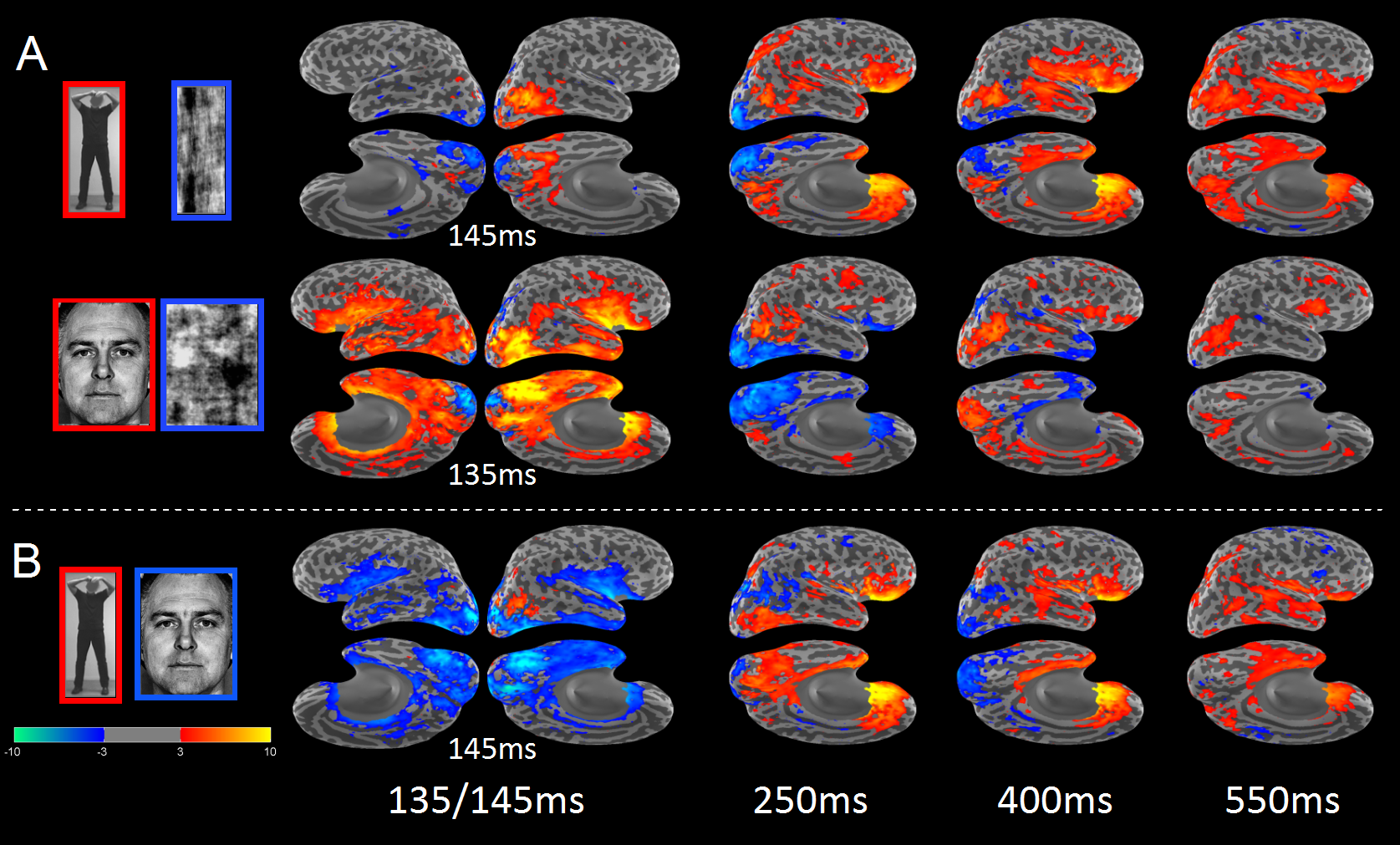

Supplement: Figure S1 — Differential maps of cortical source distribution. Group (n = 10 subjects) results of the anatomically constrained distributed source analysis (dSPM) for Face and Body perception, visualized on the inflated cortical surface at different time instants. The first column shows the source distribution at the latency of the M140 peak response in the lateral posterior sensors (135 ms for Faces, 145 ms for Bodies). The second, third and fourth column show the source distribution at the latencies of GFP maxima for bodies at 250 ms, 400 ms and 550 ms. A. Regions of Body- and Face-sensitivity were explored by subtracting the dSPM values of the Scrambled bodies and faces from the dSPM values of intact Bodies and Faces. B. Differential dSPM maps were created by contrasting the Body and Face dSPM maps directly to each other to explore regions showing category-preferred responses. The visualization of the cortical surface is identical to that in Figure 1 of the main article. The face image was taken from Ekman & Friesen [94]. (TIF) [file pone.0071408.s001.tif]

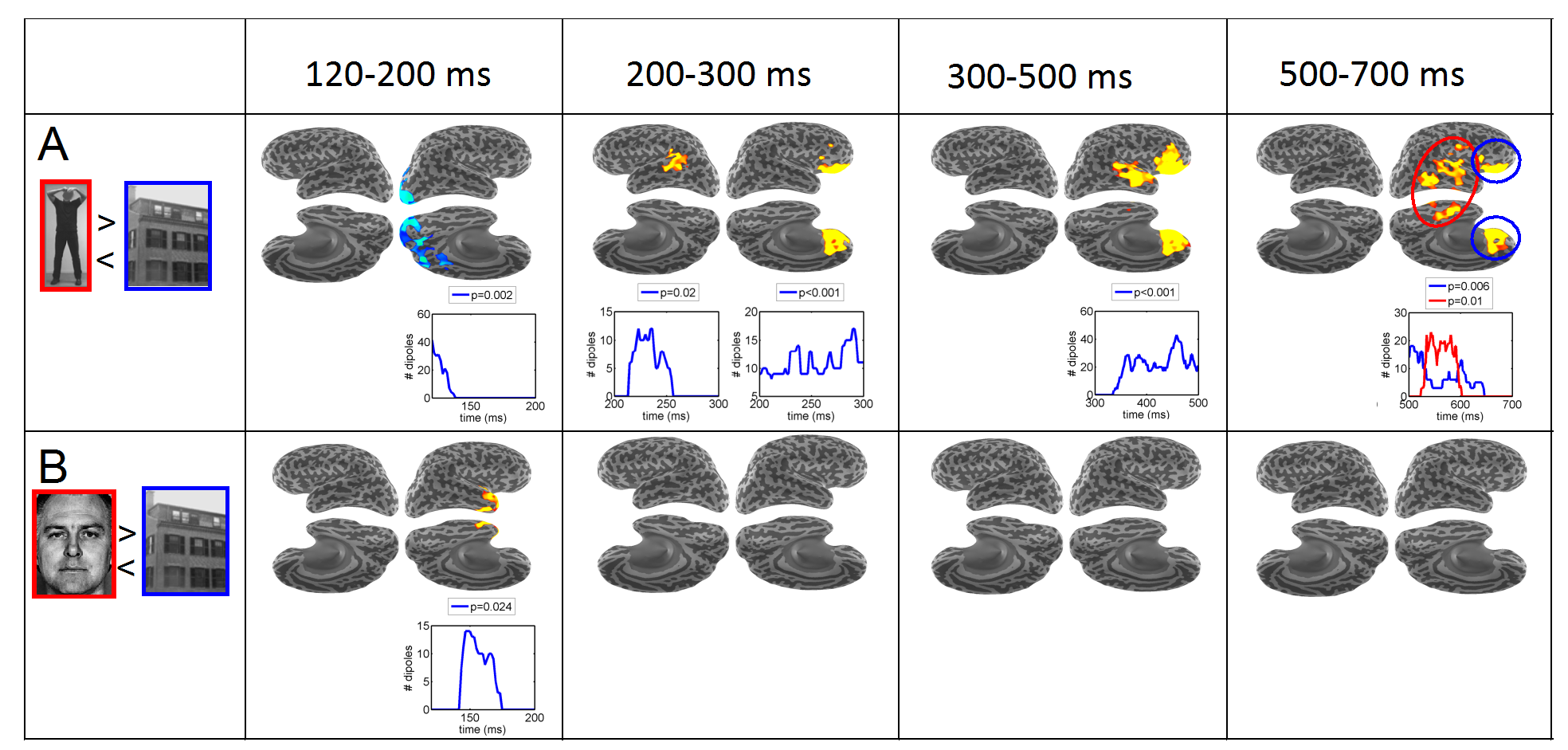

Supplement: Figure S2 — Spatiotemporal cluster analysis of cortical current estimates with respect to Houses. Results of the spatiotemporal cluster analysis on the cortical current estimates data for Face and Body perception, visualized on the inflated cortical surface. Columns represent different time windows, rows different contrasts. Each cell displays both the spatial and temporal extent of each cluster. The visualization of the inflated cortical surface is equivalent to Figure 3 (see figure legend 3D), with left hemisphere on the left and right hemisphere on the right. The graphs below the cortical maps represent the temporal courses of the size of each cluster (in number of dipoles) and their p-value, in the left and right hemisphere. In the case of multiple clusters, the red, blue and green colors indicate corresponding clusters in the cortical map (circles) and time course. A. Body selectivity was analysed by directly contrasting Bodies with Houses (n = 10, two-sided, α = 0.025 for each side). Clusters with preferred responses to Bodies are indicated in yellow/red; clusters with preferred responses to Houses in blue. B. Face selectivity was analysed by directly contrasting Faces with Houses (n = 10, two sided, α = 0.025 for each side). Clusters with preferred responses to Faces are indicated in yellow/red; clusters with preferred responses to Houses in blue. The face image was taken from Ekman & Friesen [94]. (TIF) [file pone.0071408.s002.tif]

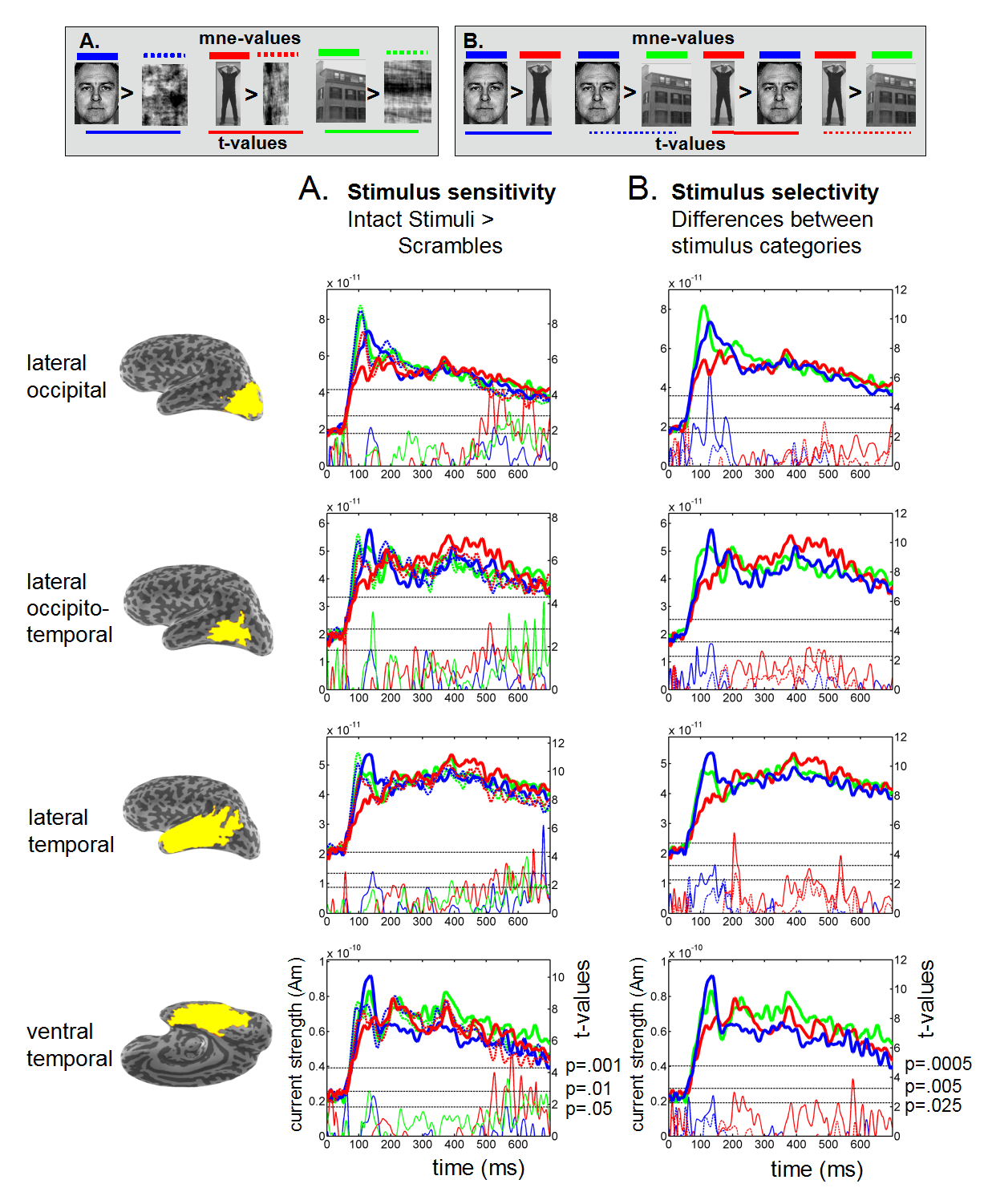

Supplement: Figure S3 — Time courses of MEG source estimates in anatomical regions of interest from the left hemisphere. Grand average (n = 10 subjects) time courses of the mean estimated current strength (thick lines) for intact (solid lines) Faces (blue), Bodies (red) and Houses (green) and their Fourier-scrambled versions (dashed lines), extracted from several large cortical regions. The thin line curves show the corresponding t-values for planned comparisons. The dotted black horizontal lines indicate the t-thresholds that correspond to α-values of 0.05, 0.01 and 0.001. A. Category sensitivity: The thin lines display the t-values for the contrasts between each intact stimulus category and its own scrambled counterpart (paired t-tests, one-sided, n = 10 subjects, df = 9) in blue (Faces>Scrambled Faces), red (Bodies>Scrambled Bodies) and green (Houses>Scrambled Houses). The dotted black horizontal lines indicate the t-thresholds that correspond to p-values of 0.05, 0.01 and 0.001. B. Category selectivity: The thin lines represent the t-values for the following contrasts: Faces>Bodies (blue solid line), Faces>Houses (blue dotted line), Bodies>Faces (red solid line) and Bodies>Houses (red dotted line). The contrasts were tested two-sided (n = 10, df = 9), but only one side is presented in the graph. Consequently, the p-values correspond to α/2. The dotted black horizontal lines indicate the t-thresholds that correspond to p-values of 0.025, 0.005 and 0.0005. Note that the vertical scales on the left axis for mne-values, and on the right axis for the t-values vary between graphs. The face image was taken from Ekman & Friesen [94]. (TIF) [file pone.0071408.s003.tif]
